# Polar Lipids of Marine Microalgae *Nannochloropsis oceanica* and *Chlorococcum amblystomatis* Mitigate the LPS-Induced Pro-Inflammatory Response in Macrophages

**DOI:** 10.3390/md21120629

**Published:** 2023-12-06

**Authors:** Tiago Conde, Bruno Neves, Daniela Couto, Tânia Melo, Diana Lopes, Rita Pais, Joana Batista, Helena Cardoso, Joana Laranjeira Silva, Pedro Domingues, M. Rosário Domingues

**Affiliations:** 1 Mass Spectrometry Centre, LAQV-REQUIMTE, Department of Chemistry, University of Aveiro, Santiago University Campus, 3810-193 Aveiro, Portugal; danielacouto@ua.pt (D.C.); taniamelo@ua.pt (T.M.); ana.s.pais@ua.pt (R.P.); joanabatista28@ua.pt (J.B.); 2 CESAM—Centre for Environmental and Marine Studies, Department of Chemistry, University of Aveiro, Santiago University Campus, 3810-193 Aveiro, Portugal; dianasalzedaslopes@ua.pt (D.L.); p.domingues@ua.pt (P.D.); 3 Department of Medical Sciences and Institute of Biomedicine—iBiMED, University of Aveiro, 3810-193 Aveiro, Portugal; bruno.neves@ua.pt; 4 R&D Department, Allmicroalgae—Natural Products S.A., Rua 25 de Abril s/n, 2445-413 Pataias, Portugal; helena.cardoso@allmicroalgae.com (H.C.); joana.g.silva@allmicroalgae.com (J.L.S.)

**Keywords:** microalgae, *Nannochloropsis oceanica*, *Chlorococcum amblystomatis*, lipids, lipidomics, betaine lipids, glycolipids, phospholipids, anti-inflammatory activity

## Abstract

Microalgae are recognized as a relevant source of bioactive compounds. Among these bioactive products, lipids, mainly glycolipids, have been shown to present immunomodulatory properties with the potential to mitigate chronic inflammation. This study aimed to evaluate the anti-inflammatory effect of polar lipids isolated from *Nannochloropsis oceanica* and *Chlorococcum amblystomatis*. Three fractions enriched in (1) digalactosyldiacylglycerol (DGDG) and sulfoquinovosyldiacylglycerol (SQDG), (2) monogalactosyldiacylglycerol (MGDG), and (3) diacylglyceryl-trimethylhomoserine (DGTS) and phospholipids (PL) were obtained from the total lipid extracts (TE) of *N. oceanica* and *C. amblystomatis*, and their anti-inflammatory effect was assessed by analyzing their capacity to counteract nitric oxide (NO) production and transcription of pro-inflammatory genes *Nos2*, *Ptgs2*, *Tnfa*, and *Il1b* in lipopolysaccharide (LPS)-activated macrophages. For both microalgae, TE and Fractions 1 and 3 strongly inhibited NO production, although to different extents. A strong reduction in the LPS-induced transcription of *Nos2*, *Ptgs2*, *Tnfa*, and *Il1b* was observed for *N. oceanica* and *C. amblystomatis* lipids. The most active fractions were the DGTS-and-PL-enriched fraction from *N. oceanica* and the DGDG-and-SQDG-enriched fraction from *C. amblystomatis*. Our results reveal that microalgae lipids have strong anti-inflammatory capacity and may be explored as functional ingredients or nutraceuticals, offering a natural solution to tackle chronic inflammation-associated diseases.

## 1. Introduction

Algae have long been used in traditional medicine and are gaining interest worldwide as a source of bioactive compounds [1,2,3,4], namely due to their capacity to attenuate inflammation associated with different diseases [5]. Inflammation is a physiological response of the host to injury or infection. However, if imbalanced, it can develop into a persistent low-grade chronic status, damaging cells, tissues, and organs, ultimately instigating the onset of chronic diseases such as cancer, cardiovascular diseases, and diabetes, among others [6]. These noncommunicable diseases (NCD) are the leading causes of death globally, accounting for around 74% of deaths, according to the World Health Organization [7].

The treatment of inflammation majorly relies on non-steroidal anti-inflammatory drugs, which are often associated with severe adverse effects such as gastrointestinal bleeding, nephrotoxicity, and cardiovascular complications [8]. Thus, natural sources have been explored as an alternative to finding new anti-inflammatory products to tackle chronic inflammation without the risk of severe side effects [9]. Natural products represent a source of many bioactive compounds for functional foods and pharmaceutical applications and have long been used in traditional medicine [10]. The use of natural products also copes with the growing concern over the negative impact of synthetic bioactive products on the environment (e.g., chemical pollution and Climate Change) increasing the pursuit of natural sources of bioactive extracts and/or compounds.

Algae, macro- and microalgae, are recognized as natural reservoirs of diverse bioactive molecules, such as carbohydrates, proteins, minerals, polyunsaturated fatty acids (PUFA), pigments, and phycobilins [11]. Microalgae are being highlighted as rich and sustainable natural sources of anti-inflammatory compounds, including lipids, such as omega-3 fatty acids, which are well-known precursors of pro-resolving mediators [12], and, more recently, polar lipids [2,5]. Microalgae fractions rich in different polar lipids, such as the betaine class of diacylglyceryltrimethylhomoserine (DGTS) species and the glycolipid classes of monogalactosyldiacylglycerol (MGDG) and digalactosyldiacylglycerol (DGDG), from *Nannochloropsis granulata* and *Tetraselmis chui*, were shown to cause a strong inhibition of lipopolysaccharide (LPS)-triggered pro-inflammatory status in Raw264.7 macrophages [13,14,15]. However, the anti-inflammatory potential of only a handful of microalgae has been explored, which is often characterized by the use of complex lipid extracts [5], hindering the understanding of the relation between the lipid structure and anti-inflammatory activity as well as the selection of the most bioactive lipid or lipid class.

To further understand the anti-inflammatory potential of polar lipids from two eicosapentaenoic acid (EPA)-rich microalgae, *Chlorococcum amblystomatis* and *Nannochloropsis oceanica*, their total lipid extracts (TE) were fractionated into glycolipids, betaines, and phospholipid-enriched fractions using solid-phase extraction (SPE). The anti-inflammatory activity was evaluated in LPS-stimulated macrophages through the assessment of NO production and the transcription levels of nitric oxide synthase (*Nos2*), cyclooxygenase-2 (*Ptgs2*), tumor necrosis factor-α (*Tnfa*), and interleukin-1β (I*l1b*).

## 2. Results

### 2.1. Composition of Lipid Extracts and Fractions from Nannochloropsis oceanica and Chlorococcum amblystomatis

**Figure 1:**
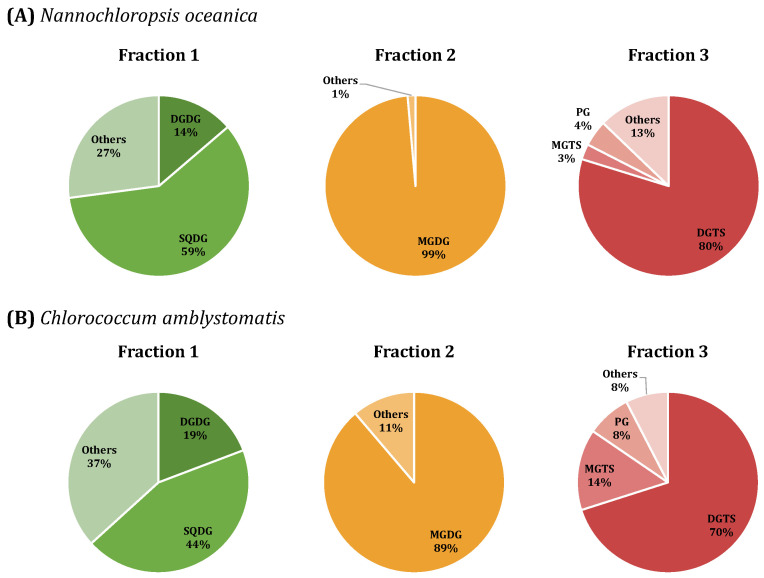
Composition of fractions 1–3 obtained after solid-phase extraction of total extracts (TE) of the microalgae *Nannochloropsis oceanica* (**A**) and *Chlorococcum amblystomatis* (**B**). Fraction 1 was mainly composed of digalactosyldiacylglycerol (DGDG) and sulfoquinovosyldiacylglycerol (SQDG) species, fraction 2 was constituted mainly by monogalactosyldiacylglycerol (MGDG) species, and fraction 3 was majorly composed of diacylglyceryltrimethylhomoserine (DGTS), monoacylglyceryltrimethylhomoserine (MGTS), and phosphatidylglycerol (PG) species.

The total lipid extracts obtained for *N. oceanica* and *C. amblystomatis* were separated into three different fractions enriched in different classes of polar lipids. Fraction 1 was enriched in DGDG and SQDG lipids, Fraction 2 was enriched in MGDG lipids, and Fraction 3 was enriched in PL and DGTS lipids, as described in Figure 1.

The lipid composition of each fraction was confirmed via RP-HPLC-MS and MS/MS, and the polar lipids identified for each alga and each class were in accordance with the lipidome identification from these microalgae previously reported (Supplementary Table S1) [16,17,18]. The most abundant lipid species from the major classes identified in each fraction are described in Table 1. The SPE allowed to obtainof fractions for each alga enriched in the same most abundant lipid classes, as observed in Figure 1, although with different compositions of lipid species.

### 2.2. Impact of Nannochloropsis oceanica and Chlorococcum amblystomatis Extracts and Fractions on Raw 264.7 Cell Viability

**Figure 2:**
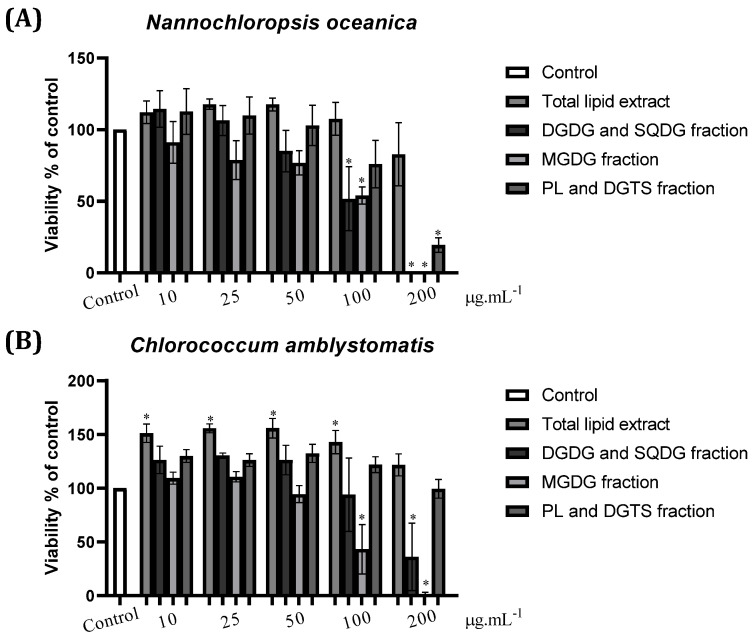
Effect of (**A**) *Nannochloropsis oceanica* and (**B**) *Chlorococcum amblystomatis* total lipid extracts and three fractions enriched in lipids from the DGDG and SQDG, MGDG, and PL and DGTS classes, respectively, on the murine cell line Raw 264.7 viability. Cells were treated with total lipid extracts and fractions at concentrations of 10, 25, 50, 100, and 200 µg·mL^−1^ from (**A**) *N. oceanica* or (**B**) *C. amblystomatis* for 24 h. Cell viability is expressed as a percentage of resazurin reduction in comparison to control cells (100% viability). Each value represents the mean ± standard deviation of three independent experiments performed in duplicate. Statistical differences between groups were calculated using a One-way ANOVA followed by Dunnet’s post hoc test (* *p* < 0.05).

The effect of *N. oceanica* and *C. amblystomatis* total lipid extracts and fractions on the viability of Raw 264.7 cells was assessed using the resazurin reduction assay (Figure 2), allowing disclosure of safe concentrations for further use. Note that in this work, we considered decreases in cell viability down to 80% of viable cells. The results showed that total lipid extracts did not affect the viability of Raw 264.7 cells at any concentration for both microalgae. The DGDG-and-SQDG-enriched fraction from *N. oceanica* affected cell viability at 50, 100, and 200 µg·mL^−1^, while the MGDG-enriched fraction affected cell viability at and beyond 25 µg·mL^−1^. The *N. oceanica* fraction enriched in PL and DGTS affected cell viability at 100 and 200 µg·mL^−1^. On the other hand, *C. amblystomatis* fractions only affected macrophage viability at 100 and 200 µg·mL^−1^.

For *N. oceanica*, the concentrations chosen to carry on with the study were 100 µg·mL^−1^ of TE, 25 µg·mL^−1^ of DGDG-and-SQDG-enriched fraction, 10 µg·mL^−1^ of MGDG-enriched fraction, and 50 µg·mL^−1^ of PL-and-DGTS-enriched fractions. In the case of *C. amblystomatis*, the selected concentrations were 100 µg·mL^−1^ for TE and 50 µg·mL^−1^ for all three enriched fractions.

### 2.3. Antioxidant Potential of Nannochloropsis oceanica and Chlorococcum amblystomatis Lipid Extracts and Fractions

**Figure 3:**
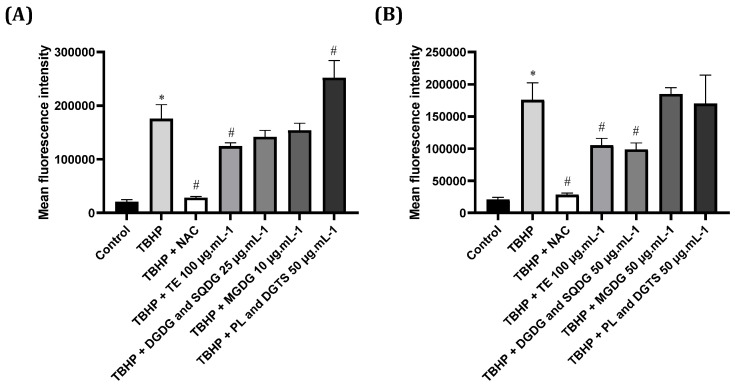
Antioxidant potential of (**A**) *Nannochloropsis oceanica* and (**B**) *Chlorococcum amblystomatis* lipid extracts and fractions against THBP-induced production of reactive oxygen species (ROS) in Jurkat cells. N-acetyl-cysteine (NAC) was used as a positive control against THBP-induced production of ROS. Values represent the mean ± standard deviation of three independent experiments performed in duplicate. Statistical differences between control and THBP groups (*) and treatment conditions and THBP (#) were evaluated using One-way ANOVA followed by Dunnet’s post hoc test (*p* < 0.05).

The antioxidant potential of microalgae lipid extracts and fractions towards ROS production was assessed (Figure 3). Firstly, the pro-oxidant stimulus TBHP induced a significant increase in ROS production, which was strongly inhibited by the classical antioxidant compound NAC. In what concerns microalgae lipid extracts and fractions, ROS production was significantly reduced when using 100 µg·mL^−1^ total lipid extracts of *N. oceanica* or *C. amblystomatis*. Moreover, the fraction enriched in DGDG and SQDG from *C. amblystomatis* also promoted a significant decrease in the TBHP-induced production of ROS. Curiously, the fraction enriched in PL and DGTS from *N. oceanica* significantly enhanced the TBHP-induced production of ROS.

### 2.4. Effect of Nannochloropsis oceanica and Chlorococcum amblystomatis Lipid Extracts and Fractions on LPS-Triggered Nitric Oxide Production via Macrophages

**Figure 4:**
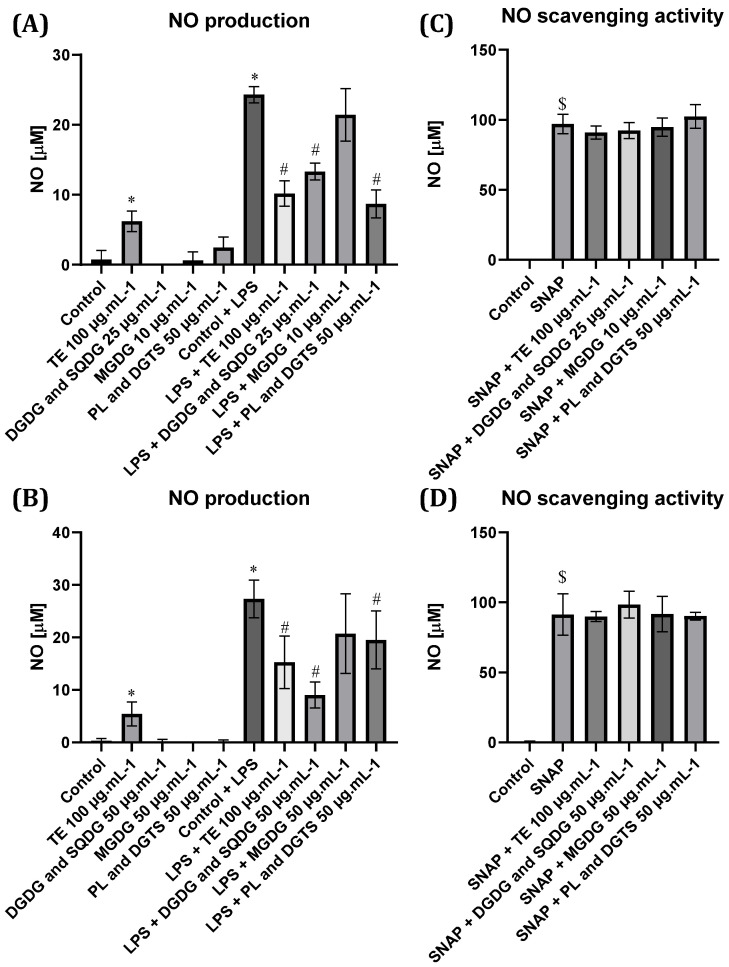
Nitrite (NO) production in Raw264.7 cells treated with (**A**) *Nannochloropsis oceanica* and (**B**) *Chlorococcum amblystomatis* lipid extracts and fractions. Effect of (**C**) *N. oceanica* and (**D**) *C. amblystomatis* lipid extracts and fractions on NO scavenging. Each value represents the mean ± standard deviation of three experiments performed in duplicate. Statistical differences between control and LPS or treatment groups (*), treatment condition and LPS (#), and control and SNAP ($) were analyzed using One-way ANOVA followed by Dunnet’s post hoc test (*p* < 0.05).

To assess the anti-inflammatory potential of *N. oceanica* and *C. amblystomatis* lipids, their impact on the production of LPS-triggered NO was evaluated in Raw 264.7 macrophages (Figure 4A,B). As expected, untreated cells had very low production of NO, as well as when co-cultured with the three different fractions from each of the microalgae. However, a significant increase in NO levels was detected when treating cells with total lipid extracts from both *N. oceanica* and *C. amblystomatis*.

LPS stimulation of Raw 264.7 macrophages induced a significant increase in the production of NO (27.3 ± 3.1 µM). Pre-treatment of cells with total lipid extracts from *N. oceanica* and *C. amblystomatis* strongly reduced LPS-induced production of NO, and the same trend was observed when using the three lipid fractions enriched in (1) DGDG and SQDG and (2) PL and DGTS from each microalga. The MGDG-enriched fraction from each microalga did not promote any significant decreases in the production of NO.

To address if the observed effects were resulting from intrinsic NO scavenging activity of the lipids, an in chemico assay was performed using SNAP as a NO donor. As shown in Figure 4C,D, neither lipid extracts nor fractions presented significant NO scavenging activity. Thus, the observed decrease in NO levels may occur through downmodulation of *Nos2* transcription and/or direct inhibition of iNOS enzymatic activity.

### 2.5. In Chemico Determination of Nannochloropsis oceanica and Chlorococcum amblystomatis Lipid Extracts and Fractions in COX-2 Activity

**Figure 5:**
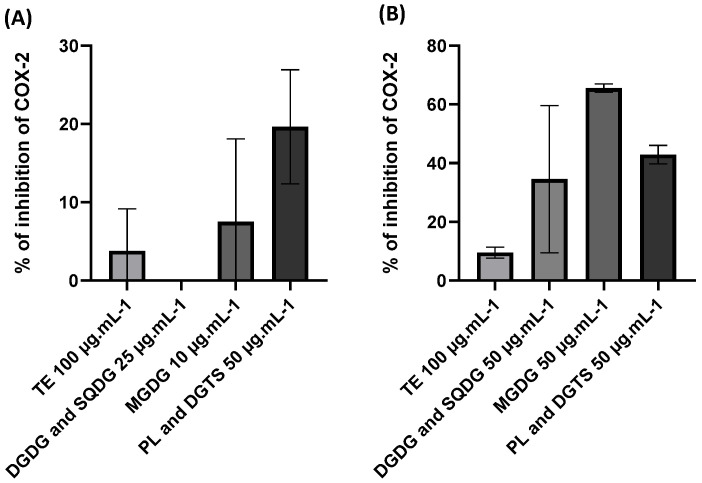
Inhibition of cyclooxygenase-2 (COX-2) activity *in chemico* using *Nannochloropsis oceanica* (**A**) and *Chlorococcum amblystomatis* (**B**) total extracts (TE) and fractions enriched in DGDG and SQDG, MGDG, and PL and DGTS. Each value represents the mean ± standard deviation of three independent experiments.

The anti-inflammatory potential of microalgae lipid fractions and extracts was further assessed through an *in chemico* COX-2 inhibition assay (Figure 5). Incubation of COX-2 with *N. oceanica* total lipid extracts and fractions of DGDG and SQDG as well as MGDG showed virtually no inhibition of the enzyme, while PL and DGTS fractions achieved 20% inhibition. On the other hand, *C. amblystomatis* total lipid extracts and fractions achieved some degree of inhibition, with the most active fraction being the MGDG fraction, which registered over 60% inhibition.

### 2.6. Effect of Nannochloropsis oceanica and Chlorococcum amblystomatis Lipid Extracts and Fractions on LPS-Triggered Transcription of Nos2, Ptgs2, Tnfa, and Il1b Genes

**Figure 6:**
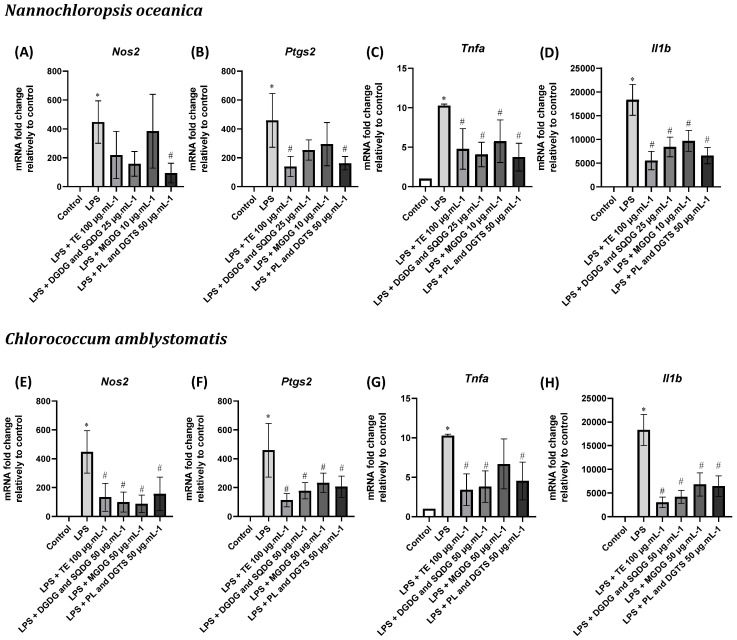
Modulation of LPS-induced transcription of pro-inflammatory genes (*Nos2*, *Ptgs2*, *Tnfa*, and *Il1b*) using *Nannochloropsis oceanica* (**A**–**D**) and *Chlorococcum amblystomatis* (**E**–**H**) lipid extracts (TE) and fractions (DGDG and SQDG, MGDG, and PL and DGTS) in Raw 264.7 cells. The mRNA levels were assessed with quantitative Real-Time RT-PCR. Results are presented as fold change relative to control and normalized with *Hprt1* as a housekeeping gene. Each value represents the mean ± standard deviation from three independent biological experiments. Statistical differences between control and LPS-stimulated cells (*) and treatment condition and LPS (#) were evaluated using One-way ANOVA followed by Dunnet’s post hoc test (*p* < 0.05).

The impact of microalgae lipids and extracts on the transcription of the pro-inflammatory genes *Nos2*, *Ptgs2*, *Tnfa*, and *Il1b* was evaluated in cells treated with microalgae and with LPS (Figure 6). As expected, treatment with LPS increased the mRNA levels of all genes analyzed.

Pre-treatment with *N. oceanica* total lipid extract significantly counteracted LPS-induced levels of *Ptgs2*, *Tnfa*, and *Il1b*. Fractions enriched in DGDG and SQDG as well as MGDG only significantly reduced pro-inflammatory cytokine genes but not *Ptgs2* and *Nos2*, while PL-and-DGTS-enriched fractions down-regulated all analyzed genes. The latter represented, therefore, the fraction with the most significant inhibition for all genes.

On the other hand, lipid extract and fractions from *C. amblystomatis* reduced the mRNA of all four genes, except for the MGDG-enriched fraction at 50 µg·mL^−1^ against *Tnfa*. The fraction enriched in DGDG and SQDG promoted the most significant decrease in transcription of all analyzed genes.

## 3. Discussion

Multiple products of natural origin have been described as having strong anti-inflammatory effects, including microalgae extracts and lipids [5]. This work explored the anti-inflammatory potential of two EPA-rich microalgae, *N. oceanica* and *C. amblystomatis*. The anti-inflammatory effect of both microalgae extracts was assessed through their capacity to mitigate cellular oxidative stress and LPS-triggered production of NO and transcription of inflammation-related genes *Nos2*, *Ptgs2*, *Tnfa*, and *IL1b*.

The total extracts from both *N. oceanica* and *C. amblystomatis* showed strong antioxidant activity and great capacity to counteract LPS-induced production of NO and transcription of the above-mentioned mediators/effectors when using non-cytotoxic concentrations. This is in line with previous work performed with both of these microalgae, which verified anti-inflammatory potential in *N. oceanica* ethanolic extract through inhibition of iNOS and COX-2 LPS-induced expression in a mouse model [19] and inhibition of COX-2 in chemico using lipid extracts from *C. amblystomatis* [18]. The influence of these two microalgae extracts on ROS production and cytokine expression has not been previously assessed. However, inhibition of LPS-induced secretion of TNF-α and IL-1β was observed for *Spirulina maxima*, *Chlorella vulgaris*, and *Phaeodactylum tricornutum* [20,21,22], while inhibition of ROS generation was observed when using *Porphyridium cruentum* extracts [23]. However, lipid extracts are complex mixtures of lipids, including polar lipids, such as phospholipids and glycolipids. In the case of *N. oceanica* and *C. amblystomatis*, the lipids were characterized, revealing a dissimilar profile, but both contained several polar lipids esterified to EPA [16,18]. To determine the lipid classes that mostly contributed to the observed anti-inflammatory activity of the extracts of *N. oceanica* and *C. amblystomatis*, we obtained, via SPE, three different fractions enriched in (1) DGDG and SQDG, (2) MGDG, and (3) PL and DGTS.

Our results revealed that only the GL fraction (DGDG and SQDG) from *C. amblystomatis* significantly reduced TBHP-induced ROS levels in lymphocytes. Inflammation-related disorders are multifactorial, and oxidative stress is an important inducer and promoter of inflammation, regulating, for instance, the NF-κB signaling pathway [24]. Moreover, ROS over and/or continuous production can cause dysfunction and tissue injury at the site of inflammation and have a negative impact on immune cells, such as T-cells [25], contributing to the onset of chronic inflammation, which is implicated in the pathogenesis of non-communicable diseases (e.g., cardiovascular disease) [26]. Modulating ROS production can prevent further aggravation of chronic inflammation and ameliorate conditions associated with high ROS levels, such as in CVD and diabetes [27]. Curiously, there was a significant increase in ROS production upon treatment of THBP-induced cells with the *N. oceanica* fraction enriched in DGTS and PL. This result can be interpreted as a potential enhancement of the THBP pro-oxidant effect on these cells. In fact, betaine lipids have been shown to increase mitochondrial respiration, which can positively affect ROS production [28]. However, future work is necessary to understand the observed changes.

Another free radical involved in inflammation that is found elevated during the pro-inflammatory phase and in chronic inflammation is NO [29]. Except for the MGDG fraction, all fractions from *N. oceanica* and *C. amblystomatis* showed strong inhibition of LPS-induced production of NO when using non-cytotoxic concentrations. A similar trend was observed when assessing *Nos2* mRNA levels. Since no scavenging of NO was observed, this indicates that the observed effects of these microalgae lipids were due in part to the decreased expression of the enzyme. However, we cannot discard the fact that lipids also directly inhibit iNOS activity. NO is a versatile free radical that can act as a neurotransmitter, vasodilator, and in the immune defense of the host [29]. Nevertheless, excessive and prolonged production of NO can promote mitochondrial dysfunction and cell apoptosis and contribute to the pathogenesis of inflammatory diseases and cancer [30]. Some studies also reported an association between high NO levels and several prevalent neurodegenerative diseases, including Parkinson’s disease, Alzheimer’s disease, amyotrophic lateral sclerosis, Huntington’s disease, and ischemic brain injury stroke [29]. In rheumatoid arthritis, elevated NO is often considered a disease marker [31], and pharmacological interventions envisioning its decrease are shown to be a valid therapeutic approach [32].

The most active fractions in the reduction in NO and *Nos2* transcription were the PL-and-DGTS-enriched fraction from *N. oceanica* and the fraction enriched in DGDG and SQDG from *C. amblystomatis*, not excluding the activity observed for other fractions with NO inhibitory activity. The inhibition of iNOS expression and activity was already shown for DGTS species containing EPA, such as DGTS (20:5/18:2) and DGTS (20:5/20:5), from the microalga *N. granulata* [13], and these lipid species were present in this *N. oceanica* fraction (Table 1). The iNOS inhibition was also associated with a few glycolipids, as well as several DGDG and SQDG species in microalgae, namely DGDG (16:0/18:4), DGDG (20:5/18:2), DGDG (20:5/20:5), and SQDG (18:3/16:0) [14,33,34], which were also identified and present in the *C. amblystomatis* fraction enriched in DGDG and SQDG. This indicates that these betaine lipids and glycolipids could have had a higher contribution to the observed inhibitory activity. However, the panoply of mediators involved in inflammation can still contribute to the aggravation of chronic inflammation, and having only one specific target might not be enough to attenuate and promote the resolution of inflammation. Therefore, it is necessary to assess the impact of these fractions on other targets.

The LPS-induced transcription of *Ptgs2*, the gene coding for cyclo-oxygenase 2 (COX-2), was strongly inhibited with the *N. oceanica* and *C. amblystomatis* fractions, excluding the MGDG fraction from *N. oceanica*. Regarding the effects directly on the enzyme activity, we observed, in chemico, little to no inhibition of COX-2 activity when using *N. oceanica* extracts, while the most active fraction of *C. amblystomatis* was enriched in MGDG. COX-2 is the inducible form of cyclo-oxygenase and catalyzes the conversion of ARA to prostaglandins. Prostaglandins are involved in multiple physiological and pathophysiological processes such as platelet aggregation, vascular permeability, thrombus formation, inflammatory response, and tumorigenesis [35,36]. Moreover, in obesity and diabetes, adipose tissue COX-2 activation contributes to fat inflammation and insulin resistance [37]. Interestingly, inhibition of COX-2 can significantly attenuate inflammation in the adipose tissue through suppression of MCP-1 and TNF-α gene expression, thus highlighting the beneficial role of targeting COX-2 to control inflammatory diseases and non-communicable diseases [38]. Nowadays, the basis of anti-inflammatory pharmacotherapy is based on COX (1/2) inhibition [39]. However, treatment with most synthetic COX inhibitors is associated with gastrointestinal damage (COX-1), and the few that have a reduced gastrointestinal impact have been associated with increased cardiovascular risk (COX-2) [40]. Thus, new alternatives are sought after, and the use of microalgae lipids offers a new natural and alternative source of compounds that can act as COX inhibitors.

The most bioactive fractions from *N. oceanica* and *C. amblystomatis* were the ones enriched in PL and DGTS, and DGDG and SQDG, respectively, which suggests these lipid classes could have had a higher contribution to the extracts’ activity. Although we did not evaluate prostaglandin levels, down-regulation of COX-2 transcription via microalgal lipids expectably affected their production, as previously reported [41]. Moreover, in the case of COX-2, no work has attributed inhibitory activity to individual lipid species, which should be explored in the future to understand the relationship between structure and COX-2 inhibitory activity.

Pre-treatment of macrophages with all lipid fractions decreased LPS-induced mRNA levels of the cytokines TNF-α and IL-1β, with the exception of the MGDG-enriched fraction from *C. amblystomatis*. TNF-α and IL-1β are two pro-inflammatory cytokines that contribute to the initiation and progression of inflammation by inducing the secretion of other pro-inflammatory cytokines and mediators, some of which are associated with pain, swelling, and tissue damage [42,43]. Overproduction and prolonged exposure to these cytokines can lead to tissue injury, sepsis, and death [44]. They are strongly implicated in the pathogenesis of inflammatory diseases like rheumatoid arthritis, atherosclerosis [45], and diabetes [46]. Interestingly, previous work with DGTS-enriched extracts from the soil alga *Chromochloris zofingiensis* and DGDG species isolated from *Iscochrysis galbana* and from the cyanobacterium *Nodularia harveyana* showed strong inhibitory activity against TNF- α secretion [47,48,49]. Thus, this corroborates the strong inhibitory power of these lipids against these cytokines.

The mechanisms of action of microalgae lipids against pro-inflammatory mediators remain underexplored. EPA is often described as being highly present in algae of marine origin [50], such as *N. oceanica* [51]. On the other hand, *C. amblystomatis* is a freshwater microalga with a considerable amount of EPA (~9%) [18]. EPA is often associated with anti-inflammatory activity, representing an important precursor of anti-inflammatory lipid mediators [52]. In microalgae, omega-3 PUFA is often found esterified in glycolipids and betaine lipids. These lipids also have intrinsic bioactive properties in addition to being carriers of omega FA [53]. For instance, Banskota et al. have addressed the impact of isolated DGTS, MGDG, and DGDG lipids against LPS-induced NO production via macrophages, observing a reduction in NO levels [13,14]. This reduction was not associated with NO scavenging but with down-regulation of iNOS expression, similar to our results, and a consequent decrease in iNOS protein levels. Curiously, in the case of DGTS isolated from the marine microalgae *N. granulate*, the NO inhibitory activity was significantly higher in DGTS (20:5/20:5) and DGTS (20:5/20:4) when compared to DGTS esterified with other FAs. However, all tested DGTS significantly decreased NO production, thus indicating that inhibition was not promoted by the unsaturated FA but by the betaine lipid itself. Also DGDG esterified to EPA exhibited the capacity to reduce NO, whereas when EPA, as free FA, was tested, a much reduced NO inhibitory activity was observed when compared to polar lipids carrying EPA, indicating the intrinsic bioactive role of the polar lipid as DGDG and MGDG [33]. On the other hand, the relationship between betaine lipids and glycolipids and their anti-inflammatory activity against other mediators remains unknown. The results herein indicate that inhibition of COX-2, TNF-α, and IL-1β can occur through the reduction in *Ptgs2*, *Tnfa*, and *Il1b*. However, further studies are necessary to precisely define this relationship.

Regulation of these pro-inflammatory mediators is associated with the signaling cascade of NF-κB, which is translocated to the cell nucleus in response to pro-inflammatory stimuli like LPS, inducing expression of *Nos2*, *Ptgs2*, *Tnfa*, *Il1b*, and others [54,55]. The inhibitory effect observed for *N. oceanica* and *C. amblystomatis* lipid fractions could arise from the suppression of this pathway, as previously reported for microalgae extracts [56]. These lipids could be interacting with receptors responsible for downstream regulation of the NF-κB pathway. However, the mechanisms of interaction between microalgae DGDG, SQDG, and DGTS remain unclear. No work has assessed the impact these lipid classes have on this signaling mediator, and future work should evaluate it. Curiously, distinct extents of inhibition were observed for the same fraction but from different microalgae at non-cytotoxic concentrations. This dissimilar activity can result from differences in the composition of lipids within the same classes [16,18]. Moreover, the MGDG-enriched fraction showed the least inhibitory strength, although previous studies observed strong inhibitory activity when using isolated MGDG from other microalgae with different lipid profiles [15,57], thus highlighting the importance of studying different microalgae as they have different compositions and combinations of lipids [17], and, in turn, different anti-inflammatory potential. While the results presented here are undoubtedly promising, further studies on in vivo models of inflammation will be of great interest for the elucidation of the pharmacokinetic behavior and, consequently, for the potential clinical applications of these extracts.

## 4. Materials and Methods

### 4.1. Reagents

HPLC-grade ethanol with 99% purity (EtOH, C_2_H_5_OH), dichloromethane (CH_2_Cl_2_), methanol (MeOH, CH_3_OH), acetone (CH_3_COCH_3_), diethyl ether (CH_3_CH_2_OCH_2_CH_3_), and acetic acid with 100% purity (CH_3_COOH) were purchased from Fisher Scientific Ltd. (Loughborough, UK) and Merck & Co., Inc. (Rahway, NJ, USA). Milli-Q water was obtained from a water purification system (Synergy, Millipore Corporation, Billerica, MA, USA). Internal standard mixture comprised 1,2-dimyristoyl-*sn*-glycero-3-phosphate (dMPA), 1,2-dimyristoyl-*sn*-glycero-3-phosphocholine (dMPC), 1-nonadecanoyl-2-hydroxy-*sn*-glycero-3-phosphocholine (LPC), 1,2-dimyristoyl-*sn*-glycero-3-phosphoethanolamine (dMPE), 1,2-dimyristoyl-*sn*-glycero-3-phospho-(10-rac-glycerol) (dMPG), 1,2-dipalmitoyl-*sn*-glycero-3-phosphatidylinositol (dPPI), 1,2-dimyristoyl-*sn*-glycero-3-phosphatidylserine (dMPS), N-heptadecanoyl-D-*erythro*-sphingosine (Cer(17:0/d18:1)), 1′,3′-bis-[1-2-di-tetradecanoyl-*sn*-glycero-3-phospho]-*sn*-glycerol (CL), and N-heptadecanoyl-D-*erythro*-sphingosylphosphorylcholine (SM). These standards were purchased from Avanti Polar Lipids, Inc. (Alabaster, AL, USA). All the other reagents and chemicals used were of the highest grade of purity commercially available.

### 4.2. Microalgal Material

Spray-dried biomasses of *Nannochloropsis oceanica* and *Chlorococcum amblystomatis* were supplied by Allmicroalgae, Natural Products S.A., located at Rua 25 de Abril s/n 2445-413 Pataias, Portugal.

Both microalgae species were cultivated autotrophically using a proprietary medium formulation based on Guillard’s F/2 [58]; saltwater strain *N. oceanica* had additional supplementation of a Magnesium mixture (Necton, Olhão, Portugal) and NaCl (Salexpor, Coimbra, Portugal) adjusted to 30 g·L^−1^ salinity. Microalgae cultures were first cultivated in 5 L flask reactors and kept in laboratory-controlled conditions: average temperature of 23 ± 1 °C and under continuous 100 μmol photons·m^2^·s^−1^ light provided using LEDs from 7 to 15 days. Five 5 L flask reactors were used to inoculate one 0.1 m^3^ L outdoor flat panel (FP) reactor, later scaled up to 1 m^3^ FPs. Four FPs were used as the inoculum of a 10 m^3^ tubular photobioreactor (PBR); these reactors were exposed to ambient light (16:8 light/dark cycles) and temperature conditions until the stationary phase was reached. A sprinkler-like irrigation system was used to keep the temperature of the PBR below the maximum limit (30 °C). pH was kept constant with pulse injections of CO_2_. The temperature limit and pH conditions in the 10 m^3^ PBRs were operated as previously described [58]. The biomass was recovered via centrifugation and dried. Microalgae at approximately 50 g·L^−1^ were dried via atomization in a spray dryer with an evaporation capacity of 150 kg water h^−1^. Drying was quickly achieved with an air stream at 215 ± 5 °C. The outlet air temperature with the biomass powder was 92 ± 3 °C. The powder was obtained through a cyclone and stored, protected from light and humidity.

### 4.3. Lipid Extraction

Lipid extraction of *C. amblystomatis* and *N. oceanica* biomass was carried out using ultrasound-assisted extraction (UAE). Ethanol 99% was added to glass tubes carrying 25 mg of biomass and vortexed for 2 min. UAE was performed using an ultrasonic water/ice bath (Bandelin, Mecklenburg-Vorpommern, Germany) for 30 min, with an ultrasound frequency of 35 kHz and a nominal ultrasonic power density of 80. The water/ice bath was used to avoid warming the medium and was renewed every 30 min. Samples were then centrifuged for 10 min at 2000 rpm, and the organic phase was collected. This procedure was repeated three more times. The combined, collected organic phases were dried under a nitrogen (N_2_) stream.

### 4.4. Solid-Phase Extraction of the Total Extracts

**Figure 7:**
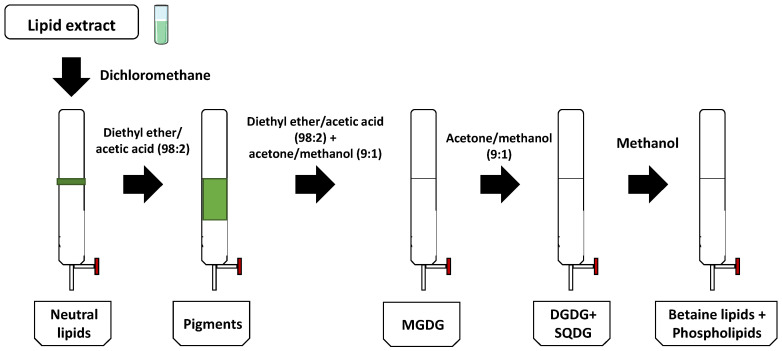
Schematic representation of solid-phase extraction to separate different lipid classes from microalgae extracts. Lipid extracts of *Nannochloropsis oceanica* and *Chlorococcum amblystomatis* were loaded into the column, and elution occurred as follows: (1) dichloromethane was used to separate neutral lipids; (2) diethylether/acetic acid (98:2 *v*/*v*) was used to separate pigments; (3) a (1:1 *v*/*v*) mixture of diethylether/acetic acid (98:2 *v*/*v*) and acetone/methanol (9:1 *v*/*v*) was used to separate MGDG; (4) acetone/methanol (9:1 *v*/*v*) was used to separate DGDG and SQDG; and (5) methanol was used to separate betaine lipids and phospholipids.

Lipid fractionation was performed according to the adapted procedure from Ruiz et al. [59,60]. Glass columns (Fisher Scientific^®^, Hampton, NH, USA) containing approximately 5 g of silica gel (Flash 40–60 μm—60 Å) (ACROS Organics^®^, Hampton, NH, USA) were used to obtain three fractions enriched in 1) DGDG and SQDG, 2) MGDG, and 3) PL and DGTS. In brief, the column was conditioned with 30 mL of dichloromethane (CH_2_Cl_2_) and loaded with 10 mg of microalgal extract dissolved in CH_2_Cl_2_. The lipid elution occurred according to Figure 7, and as follows: neutral lipids were separated and discarded using 30 mL of CH_2_Cl_2_; pigments were separated and discarded using 45 mL of a mixture of diethyl ether/acetic acid (98:2 *v*/*v*); Fraction 1 was eluted using 30 mL of a mixture (1:1 *v*/*v*) of diethyl ether/acetic acid (98:2 *v*/*v*) and acetone/methanol (9:1 *v*/*v*); Fraction 2 was eluted with 40 mL of acetone/MeOH (9:1 *v*/*v*); Fraction 3 was eluted using 40 mL of MeOH. The recovered fractions were collected and evaporated to dryness with N_2_. Silica residues were separated from the recovered fractions through an adapted Folch extraction methodology [18,61].

### 4.5. Lipidomics Analysis

#### 4.5.1. Data Acquisition

Lipid extracts and fractions were analyzed via reverse phase liquid chromatography (RP-LC) in an Ultimate 3000 Dionex (Thermo Fisher Scientific, Bremen, Germany) using an Ascentis^®^ Express C18 column (Sigma-Aldrich^®,^ St. Louis, MO, USA, 2.1 × 100 mm, 2.7 µm) coupled to the Q-Exactive^®^ hybrid quadrupole Orbitrap mass spectrometer (Thermo Fisher Scientific, Bremen, Germany). Mobile phase A was composed of water/acetonitrile (40/60%) with 10 mM ammonium formate and 0.1% formic acid. Mobile phase B was composed of isopropanol/acetonitrile (90/10%) with 10 mM ammonium formate and 0.1% formic acid. The following gradient was applied: 32% B at 0 min, 45% B at 1.5 min, 52% B at 4 min, 58% B at 5 min, 66% B at 8 min, 70% B at 11 min, 85% B at 14 min, 97% B at 18 min, 97% B at 25 min, 32% B at 25.01 min, and 32% B at 33 min.

A mixture of 40 µg of lipid extracts and fractions (in 10 µL of dichloromethane), 82 µL of a solvent system consisting of 50% isopropanol/50% methanol, and 8 µL of standards mixture (dMPC—0.04 µg, SM d18:1/17:0—0.04 µg, dMPE—0.04 µg, LPC—0.04 µg, dPPI—0.08 µg, CL (14:0)4—0.16 µg; dMPG—0.024 µg, Cer (17:0/d18:1)—0.08 μg, dMPS—0.08 µg; dMPA—0.16 μg) was prepared for each mixture, and 5 µL was loaded into the column at 50 °C and at a flow-rate of 260 µL·min^−1^. The mass spectrometer operated in simultaneous positive (ESI 3.0 kV) and negative (ESI—2.7 kV) modes, as previously described. The capillary temperature was 320 °C, and the sheath gas flow was 35 U. Data were acquired in full scan mode with a high resolution of 70,000 at *m*/*z* 200, an automatic gain control (AGC) target of 3 × 10^6^, an *m*/*z* range of 300–1600, 2 microscans, and a maximum inject time (IT) of 100 ms. The tandem mass spectra (MS/MS) were obtained with a resolution of 17,500, an AGC target of 1 × 10^5^, 1 microscan, and a maximum IT of 100 ms. The cycles consisted of one full-scan mass spectrum and ten data-dependent MS/MS scans, which were repeated continuously throughout the experiments with a dynamic exclusion of 30 s and an intensity threshold of 8 × 10^4^. Normalized collision energy TM (CE) ranged between 20, 24, and 28 eV in the negative mode and 25 and 30 eV in the positive mode. Data acquisition was performed using the Xcalibur data system (V3.3, Thermo Fisher Scientific, Bremen, Germany).

#### 4.5.2. Data Analysis

The identification of the different lipid species was based on mass accuracy observed in LC-MS spectra, as well as LC-MS/MS spectra interpretation that allows confirming the polar head group identity and the fatty acyl chains of the molecular species. MSDial 4.6 software was used for peak detection, compound identification, and the generation of a list of identified species. The generated template with the identified lipid species in the *C. amblystomatis* and *N. oceanica* extracts and fractions was further used in MZmine 2.53 software. This software was used for filtering LC-MS raw data, peak detection, peak processing, and assignment against the template generated with MSDial. Only peaks within 5 ppm of the lipid exact mass and a peak area higher than 1 × 10^7^ were considered. Relative quantification was performed by normalizing the peak areas of the extracted ion chromatograms (EIC) with the peak areas of internal standards.

### 4.6. Liposomes Preparation

The liposomes containing total extracts or lipid fractions from *C. amblystomatis* or *N. oceanica* were prepared using the modified Gortzi method [62]. The dried total extracts and fractions were mixed with sterile DMEM media, followed by two cycles of 5 min vortex and 30 min of sonication in an ultrasonic water/ice bath (Bandelin, Mecklenburg-Vorpommern, Germany) with an ultrasound frequency of 35 kHz and a nominal ultrasonic power density of 80 W, and the water/ice bath was renewed every 30 min.

### 4.7. Cell Culture

Raw 264.7 cells (ATCC No. TIB-71, Manassas, VA, USA), a mouse macrophage cell line, were maintained in high-glucose Dulbecco’s Modified Eagle Medium (DMEM) supplemented with 10% non-inactivated fetal bovine serum (FBS), 1.5 g·L^−1^ sodium bicarbonate, 2 mM of glutamine, 100 U·mL^−1^ of penicillin, and 100 µg·mL^−1^ of streptomycin. Cells were maintained in an incubator at 37 °C and 5% CO_2_ and were sub-cultured when reaching 80–90% confluence. Jurkat (ATCC No. TIB-152, Manassas, VA, USA), a human T-cell line, was cultured in RPMI 1640 (Gibco, ThermoScientific, Waltham, MA, USA) supplemented with 1 mM of pyruvate, 2 mM of glutamine, 100 U·mL^−1^ of penicillin, 100 µg·mL^−1^ of streptomycin, and 10% heat-inactivated FBS. Cells were maintained in a confluence between 0.2 and 1 × 10^6^ cells·mL^−1^. The morphological appearance of both cell lines was routinely monitored via optical microscopy.

### 4.8. Evaluation of Cell Viability Using a Resazurin Assay

The impact of lipid fractions on cell viability was determined using the resazurin reduction assay [63]. Macrophages were seeded at 5.0 × 10^5^ cells·well^−1^ in a 96-well plate and allowed to stabilize in 200 μL of media overnight in the incubator. Cells were then treated with increasing concentrations of liposomes containing total extracts (TE), DGDG and sulfoquinovosyldiacylglycerol (SQDG), MGDG, and PL-and-DGTS-enriched fractions (10, 25, 50, 100, and 200 μg·mL^−1^). After 22 h, resazurin (50 μM) was added, and cells were incubated for an additional 2 h. Absorbance was then measured at 570 and 600 nm in a Tecan infinite M200 spectrophotometer (Tecan Group, Männedorf, Switzerland). All assays were performed in biological triplicates.

### 4.9. In Vitro Antioxidant Activity

Jurkat lymphocytes were plated at 2.5 × 10^5^ cells·well^−1^ in a 24-well plate and treated with TE and fractions of both microalgae at non-cytotoxic concentrations for 1 h, followed by the addition of 200 μM of tert-butyl hydroperoxide (TBHP) for an additional hour. After this time, CellROX™ Deep Red (ThermoScientific, Waltham, MA, USA) was added to a final concentration of 500 nM, and cells were incubated for 45 min. Cells treated only with 200 μM TBHP were used as a positive control for cellular oxidative stress, and cells treated with 5 mM N-acetylcysteine (NAC) prior to TBHP addition served as a positive control for antioxidant activity. Finally, cells were collected and washed once with PBS, and the pellet was resuspended in 400 μL of FACS buffer (PBS + 2% FBS).

The samples were analyzed in an Accuri C6 with a Flow Cytometer (BD Biosciences, Franklin Lakes, NJ, USA) using the FL4 detector (filter 675/25) to measure the fluorescence of the CellROX^®^ Deep Red dye. Data were collected from at least 10,000 gated singlet events, and the results were processed using the FlowJo X software (BD Biosciences, NJ, USA).

### 4.10. Evaluation of Potential Anti-Inflammatory Activity with Blockade of LPS-Triggered NO Production

The potential anti-inflammatory activity of TE and DGDG and SQDG, MGDG, PL, and DGTS extracts was evaluated by analyzing their capacity to inhibit NO production in LPS-stimulated Raw264.7 cells. The NO accumulation as nitrite in the culture supernatants was measured using the colorimetric Griess assay, as previously described [64]. Individual plates were used for each microalga. Raw 264.7 macrophages were seeded at 4.0 × 10^5^ cells·well^−1^ in a 96-well plate and allowed to stabilize in new media for 24 h in the incubator. Cells were then incubated with *N. oceanica* TE (100 μg·mL^−1^), DGDG and SQDG (25 μg·mL^−1^), MGDG (10 μg·mL^−1^), and PL and DGTS (50 μg·mL^−1^), and *C. amblystomatis* TE (100 μg·mL^−1^), DGDG and SQDG (50 μg·mL^−1^), MGDG (50 μg·mL^−1^), and PL and DGTS (50 μg·mL^−1^), and LPS was added at a final concentration of 100 ng·mL^−1^ after 1 h of incubation. After treatment, 100 μL of supernatant and 100 μL of Griess reagent were mixed and incubated in the dark for 15 min at room temperature. The absorbance was read at 550 nm in a microplate reader (Multiskan GO 1510-00111C, ThermoScientific, Waltham, MA, USA). The quantity of nitrites was determined based on a sodium nitrite standard curve. All experiments were performed in triplicate.

### 4.11. Nitric Oxide Scavenging Potential

The NO scavenging potential of the microalgae total extracts and lipid fractions was assessed using S-nitroso-N-acetyl-DL-penicillamine (SNAP) as a NO donor. Culture medium containing *C. amblystomatis* TE (100 μg·mL^−1^), DGDG and SQDG (50 μg·mL^−1^), MGDG (50 μg·mL^−1^), and PL and DGTS (50 μg·mL^−1^), and *N. oceanica* TE (100 μg·mL^−1^), DGDG and SQDG (25 μg·mL^−1^), MGDG (10 μg·mL^−1^), and PL and DGTS (50 μg·mL^−1^), as well as SNAP (300 μM) were incubated in 96-well microplates for 3 h at 37 °C. The supernatants were collected and mixed with Griess reagent in the dark, at room temperature, for 15 min. A microplate reader (Multiskan GO 1510-00111C, ThermoScientific, Waltham, MA, USA) was used to determine the number of nitrites. All assays were performed in triplicate.

### 4.12. COX-2 Inhibition in Chemico Assay

The COX-2 inhibitory potential of *N. oceanica* and *C. amblystomatis* total lipid extracts and fractions was assessed with the COX-2 inhibition assay. This assay was performed using the commercial COX-2 inhibitory screening assay kit—Cayman test kit-701080 (Cayman Chemical Company, Ann Arbor, MI, USA)—and was carried out according to the manufacturer’s instructions. Total lipid extracts and fractions from *N. oceanica* and *C. amblystomatis* were dissolved in 100% dimethyl sulfoxide (DMSO) to the established concentrations. The amount of prostaglandin F2α was determined via spectrophotometry (415 nm, Multiskan GO 1.00.38, Thermo Scientific, Hudson, NH, USA) and processed with the software SkanIT version 3.2 (Thermo Scientific). The results were expressed as a percentage of COX-2 inhibition.

### 4.13. Analysis of Gene Transcription via Quantitative Reverse Transcription PCR (qPCR)

Raw 264.7 macrophages were plated at 2 × 10^6^ cells·well^−1^ in 6-well microplates and allowed to stabilize overnight. Then, cells were pre-incubated with microalgal lipid extract or fraction at the indicated concentrations, followed via LPS stimulation (100 ng·mL^−1^) for 24 h.

Total RNA was isolated using NZYol reagent (Nzytech, Lisboa, Portugal) according to the manufacturer’s instructions, and its concentration was determined with OD260 measurements using a Nanodrop spectrophotometer (Wilmington, DE, USA). Samples were stored in RNA Storage Solution (Ambion, Foster City, CA, USA) at −80 °C until use.

For analysis of mRNA levels of selected genes, 1 µg of total RNA was reverse transcribed using the NZY First-Strand cDNA Synthesis Kit (Nzytech, Lisboa, Portugal), and then real-time quantitative PCR (qPCR) reactions were performed using the NZYSpeedy qPCR Green Master Mix (Nzytech, Lisboa, Portugal) on a Bio-Rad CFX Connect device. Transcription levels for indicated genes were analyzed with GenEx^®^ software version 7 (MultiD Analyses AB, Göteberg, Sweden) using *Hprt1* as a reference gene and the Livak method (2-ΔΔCt), with the results expressed as fold changes relative to control. Primer sequences were designed using Beacon Designer software version 8 (Premier Biosoft International, Palo Alto, CA, USA) and thoroughly tested.

### 4.14. Statistical Analysis

The statistical analysis was carried out to identify changes between control vs. LPS-treated cells, control vs. cells incubated with microalgae extracts and lipids, and LPS-treated cells vs. cells incubated with microalgae extracts and lipids prior to LPS treatment. The software GraphPadPrism version 9 (GraphPad Software, San Diego, CA, USA) was used to compare the effects of different treatments on control or LPS-stimulated cells, and One-way or Two-way ANOVA followed by Dunnett’s multiple comparison tests with significance levels (*p* < 0.05) were used.

## 5. Conclusions

The present study demonstrated that extracts and lipid fractions from the microalgae *Nannochloropsis oceanica* and *Chlorococcum amblystomatis* suppressed pro-inflammatory-induced production of NO and transcription of *Nos2*, *Ptgs2*, *Tnfa*, and *Il1b*. The most active lipid fractions of each alga were enriched in DGTS and PL, and DGDG and SQDG, respectively. Targeting these pro-inflammatory mediators can help modulate the pro-inflammatory status and thus reduce chronic inflammation, offering these natural microalgal lipids a role as anti-inflammatory agents. Future work should explore the molecular mechanism by which these microalgal lipids exert the observed anti-inflammatory effects, and immunomodulation over other immune cells, such as dendritic cells and T cells, should also be considered.

## Figures and Tables

**Table 1:** Most abundant lipid classes in fractions 1–3 obtained from the total lipid extracts of the microalgae *Nannochloropsis oceanica* and *Chlorococcum amblystomatis*, identified via RP-LC-MS/MS. Abbreviations: DGDG—digalactosyldiacylglycerol, SQDG—sulfoquinovosyldiacylglycerol, MGDG—monogalactosyldiacylglycerol, DGTS—diacylglyceryl-trimethylhomoserine, MGTS—monoacylglyceryl-trimethylhomoserine, and PG—phosphatidylglycerol.

	*Nannochloropsis oceanica*		*Chlorococcum amblystomatis*	
	Most Abundant Lipid Classes	Most Abundant Lipid Species	Most Abundant Lipid Classes	Most Abundant Lipid Species
Fraction 1	DGDG	DGDG 16:0_16:1	DGDG	DGDG 14:0_18:1
	SQDG	SQDG 16:0_16:1	SQDG	SQDG 16:0_16:1
Fraction 2	MGDG	MGDG 20:5_20:5	MGDG	MGDG 16:0_16:1
Fraction 3	DGTS	DGTS 20:5_20:5	DGTS	DGTS 20:5_20:5
	MGTS	MGTS 20:5	MGTS	MGTS 20:5
	PG	PG 16:0_20:5	PG	PG 16:0_20:5

## Data Availability

The original data presented in the study are included in the article; further inquiries can be directed to the corresponding author.

## Supplementary Materials

The following supporting information can be downloaded at https://www.mdpi.com/article/10.3390/md21120629/s1; Table S1: Normalized extracted ion chromatograms areas of the most abundant lipid species identified in the total lipid extracts (TE) and fractions enriched in (1) digalactosyldiacylglycerol (DGDG) and sulfoquinofosyldiacylglycerol (SQDG), (2) monogalactosyldiacylglycerol (MGDG), and (3) diacylglyceryl-trimethylhomoserine (DGTS) and phospholipids (PL) from the microalga *Nannochloropsis oceanica*. Values represent an average of three independent measures and standard deviation (SD). Raw areas inferior to 10^7^ were excluded. Table S2: Normalized extracted ion chromatograms areas of the most abundant lipid species identified in the total lipid extracts (TE) and fractions enriched in (1) digalactosyldiacylglycerol (DGDG) and sulfoquinofosyldiacylglycerol (SQDG), (2) monogalactosyldiacylglycerol (MGDG), and (3) diacylglyceryl-trimethylhomoserine (DGTS) and phospholipids (PL) from the microalga *Chlorococcum amblystomatis*. Values represent an average of three independent measures and standard deviation (SD). Raw areas inferior to 10^7^ were excluded.

## References

[1] Malhotra S., Singh A.P. (2008). Algae, Traditional Medicine, and Pharmacological Advances. Int. J. Algae.

[2] Pradhan B., Nayak R., Patra S., Jit B.P., Ragusa A., Jena M. (2020). Bioactive Metabolites from Marine Algae as Potent Pharmacophores against Oxidative Stress-Associated Human Diseases: A Comprehensive Review. Molecules.

[3] Lomartire S., Gonçalves A.M.M. (2022). An Overview of Potential Seaweed-Derived Bioactive Compounds for Pharmaceutical Applications. Mar. Drugs.

[4] Silva M., Kamberovic F., Uota S.T., Kovan I.-M., Viegas C.S.B., Simes D.C., Gangadhar K.N., Varela J., Barreira L. (2022). Microalgae as Potential Sources of Bioactive Compounds for Functional Foods and Pharmaceuticals. Appl. Sci..

[5] Conde T.A., Zabetakis I., Tsoupras A., Medina I., Costa M., Silva J., Neves B., Domingues P., Domingues M.R. (2021). Microalgal Lipid Extracts Have Potential to Modulate the Inflammatory Response: A Critical Review. Int. J. Mol. Sci..

[6] Chiurchiù V., Leuti A., Maccarrone M. (2018). Bioactive Lipids and Chronic Inflammation: Managing the Fire Within. Front. Immunol..

[7] GBD 2016 Causes of Death Collaborators (2017). Global, Regional, and National Age-Sex Specifc Mortality for 264 Causes of Death, 1980–2016: A Systematic Analysis for the Global Burden of Disease Study 2016. Lancet.

[8] Gautam R., Jachak S.M. (2009). Recent Developments in Anti-Inflammatory Natural Products. Med. Res. Rev..

[9] Arulselvan P., Fard M.T., Tan W.S., Gothai S., Fakurazi S., Norhaizan M.E., Kumar S.S. (2016). Role of Antioxidants and Natural Products in Inflammation. Oxid. Med. Cell. Longev..

[10] Atanasov A.G., Zotchev S.B., Dirsch V.M., Supuran C.T., The International Natural Product Sciences Taskforce (2021). Natural Products in Drug Discovery: Advances and Opportunities. Nat. Rev. Drug Discov..

[11] Menaa F., Wijesinghe U., Thiripuranathar G., Althobaiti N.A., Albalawi A.E., Khan B.A., Menaa B. (2021). Marine Algae-Derived Bioactive Compounds: A New Wave of Nanodrugs?. Mar. Drugs.

[12] Mori T.A., Beilin L.J. (2004). Omega-3 Fatty Acids and Inflammation. Curr. Atheroscler. Rep..

[13] Banskota A.H., Stefanova R., Sperker S., McGinn P.J. (2013). New Diacylglyceryltrimethylhomoserines from the Marine Microalga Nannochloropsis Granulata and Their Nitric Oxide Inhibitory Activity. J. Appl. Phycol..

[14] Banskota A.H., Stefanova R., Gallant P., McGinn P.J. (2013). Mono- and Digalactosyldiacylglycerols: Potent Nitric Oxide Inhibitors from the Marine Microalga Nannochloropsis Granulata. J. Appl. Phycol..

[15] Banskota A.H., Gallant P., Stefanova R., Melanson R., Oleary S.J.B. (2013). Monogalactosyldiacylglycerols, Potent Nitric Oxide Inhibitors from the Marine Microalga Tetraselmis Chui. Nat. Prod. Res..

[16] Melo T., Figueiredo A.R.P., Costa E., Couto D., Silva J., Ros M., Domingues P. (2021). Ethanol Extraction of Polar Lipids from *Nannochloropsis oceanica* for Food, Feed, and Biotechnology Applications Evaluated Using Lipidomic Approaches. Mar. Drugs.

[17] Couto D., Conde T.A., Melo T., Neves B., Costa M., Silva J., Domingues R., Domingues P. (2023). The Chemodiversity of Polar Lipidomes of Microalgae from Different Taxa. Algal Res..

[18] Conde T.A., Couto D., Melo T., Costa M., Silva J., Domingues M.R., Domingues P. (2021). Polar Lipidomic Profile Shows Chlorococcum amblystomatis as a Promising Source of Value-Added Lipids. Sci. Rep..

[19] Choi J.Y., Hwang C.J., Lee H.P., Kim H.S., Han S.-B., Hong J.T. (2017). Inhibitory Effect of Ethanol Extract of *Nannochloropsis oceanica* on Lipopolysaccharide-Induced Neuroinflammation, Oxidative Stress, Amyloidogenesis and Memory Impairment. Oncotarget.

[20] Choi W.Y., Sim J.H., Lee J.Y., Kang D.H., Lee H.Y. (2019). Increased Anti-Inflammatory Effects on LPS-Induced Microglia Cells by Spirulina Maxima Extract from Ultrasonic Process. Appl. Sci..

[21] Neumann U., Louis S., Gille A., Derwenskus F., Schmid-Staiger U., Briviba K., Bischoff S.C. (2018). Anti-Inflammatory Effects of Phaeodactylum Tricornutum Extracts on Human Blood Mononuclear Cells and Murine Macrophages. J. Appl. Phycol..

[22] Sibi G., Rabina S. (2016). Inhibition of Pro-Inflammatory Mediators and Cytokines by Chlorella Vulgaris Extracts. Pharamacogn. Res..

[23] Bergé J.P., Debiton E., Dumay J., Durand P., Barthomeuf C. (2002). In Vitro Anti-Inflammatory and Anti-Proliferative Activity of Sulfolipids from the Red Alga Porphyridium Cruentum. J. Agric. Food Chem..

[24] Hussain T., Tan B., Yin Y., Blachier F., Tossou M.C.B., Rahu N. (2016). Oxidative Stress and Inflammation: What Polyphenols Can Do for Us?. Oxid. Med. Cell. Longev..

[25] Belikov A.V., Schraven B., Simeoni L. (2015). T Cells and Reactive Oxygen Species. J. Biomed. Sci..

[26] Peoples J.N., Saraf A., Ghazal N., Pham T.T., Kwong J.Q. (2019). Mitochondrial Dysfunction and Oxidative Stress in Heart Disease. Exp. Mol. Med..

[27] Ayoka T.O., Ezema B.O., Eze C.N., Nnadi C.O. (2022). Antioxidants for the Prevention and Treatment of Non-Communicable Diseases. J. Explor. Res. Pharmacol..

[28] Lee I. (2015). Betaine Is a Positive Regulator of Mitochondrial Respiration. Biochem. Biophys. Res. Commun..

[29] Knott A.B., Bossy-Wetzel E. (2009). Nitric Oxide in Health and Disease of the Nervous System. Antioxid. Redox Signal..

[30] Sharma J.N., Al-Omran A., Parvathy S.S. (2007). Role of Nitric Oxide in Inflammatory Diseases. Inflammopharmacology.

[31] Nagy G., Clark J.M., Buzás E.I., Gorman C.L., Cope A.P. (2007). Nitric Oxide, Chronic Inflammation and Autoimmunity. Immunol. Lett..

[32] Yeo J., Lee Y.M., Lee J., Park D., Kim K., Kim J., Park J., Kim W.J. (2019). Nitric Oxide-Scavenging Nanogel for Treating Rheumatoid Arthritis. Nano Lett..

[33] Banskota A.H., Stefanova R., Sperker S., Lall S.P., Craigie J.S., Hafting J.T., Critchley A.T. (2014). Polar Lipids from the Marine Macroalga Palmaria Palmata Inhibit Lipopolysaccharide-Induced Nitric Oxide Production in RAW264.7 Macrophage Cells. Phytochemistry.

[34] Bruno A., Rossi C., Marcolongo G., Di Lena A., Venzo A., Berrie C.P., Corda D. (2005). Selective in Vivo Anti-Inflammatory Action of the Galactolipid Monogalactosyldiacylglycerol. Eur. J. Pharmacol..

[35] Gandhi J., Khera L., Gaur N., Paul C., Kaul R. (2017). Role of Modulator of Inflammation Cyclooxygenase-2 in Gammaherpesvirus Mediated Tumorigenesis. Front. Microbiol..

[36] Nakanishi M., Rosenberg D.W. (2013). Multifaceted Roles of PGE2 in Inflammation and Cancer. Semin. Immunopathol..

[37] Chan P.-C., Liao M.-T., Hsieh P.-S. (2019). The Dualistic Effect of COX-2-Mediated Signaling in Obesity and Insulin Resistance. Int. J. Mol. Sci..

[38] Granado M., Martín A.I., Castillero E., López-Calderón A., Villanúa M.Á. (2009). Cyclooxygenase-2 Inhibition Reverts the Decrease in Adiponectin Levels and Attenuates the Loss of White Adipose Tissue during Chronic Inflammation. Eur. J. Pharmacol..

[39] Vonkeman H.E., van de Laar M.A.F.J. (2010). Nonsteroidal Anti-Inflammatory Drugs: Adverse Effects and Their Prevention. Semin. Arthritis Rheum..

[40] Chen W., Zhong Y., Feng N., Guo Z., Wang S., Xing D. (2021). New Horizons in the Roles and Associations of COX-2 and Novel Natural Inhibitors in Cardiovascular Diseases. Mol. Med..

[41] Lakshmegowda S.B., Rajesh S.K., Kandikattu H.K., Nallamuthu I., Khanum F. (2020). In Vitro and In Vivo Studies on Hexane Fraction of Nitzschia Palea, a Freshwater Diatom for Oxidative Damage Protective and Anti-Inflammatory Response. Rev. Bras. Farm..

[42] Zelová H., Hošek J. (2013). TNF-α Signalling and Inflammation: Interactions between Old Acquaintances. Inflamm. Res..

[43] Schenk M., Fabri M., Krutzik S.R., Lee D.J., Vu D.M., Sieling P.A., Montoya D., Liu P.T., Modlin R.L. (2014). Interleukin-1β Triggers the Differentiation of Macrophages with Enhanced Capacity to Present Mycobacterial Antigen to T Cells. Immunology.

[44] Prasad S., Aggarwal B.B. (2014). Chronic Diseases Caused by Chronic Inflammation Require Chronic Treatment: Anti-Inflammatory Role of Dietary Spices. J. Clin. Cell. Immunol..

[45] Parameswaran N., Patial S. (2010). Tumor Necrosis Factor-α Signaling in Macrophages. Crit. Rev. Eukaryot. Gene Exp..

[46] Popa C., Netea M.G., van Riel P.L.C.M., van der Meer J.W.M., Stalenhoef A.F.H. (2007). The Role of TNF-α in Chronic Inflammatory Conditions, Intermediary Metabolism, and Cardiovascular Risk. J. Lipid Res..

[47] Tena Pérez V., Apaza Ticona L., Cabanillas A.H., Maderuelo Corral S., Rosero Valencia D.F., Quintana A.M., Ortega Domenech M., Rumbero Sánchez Á. (2021). Anti-Inflammatory Activity of Glycolipids Isolated from Cyanobacterium *Nodularia Harveyana*. Nat. Prod. Res..

[48] Leitner P.D., Jakschitz T., Gstir R., Stuppner S., Perkams S., Kruus M., Trockenbacher A., Griesbeck C., Bonn G.K., Huber L.A. (2022). Anti-Inflammatory Extract from Soil Algae Chromochloris Zofingiensis Targeting TNFR/NF-κB Signaling at Different Levels. Cells.

[49] De Los Reyes C., Ortega M.J., Rodríguez-Luna A., Talero E., Motilva V., Zubía E. (2016). Molecular Characterization and Anti-Inflammatory Activity of Galactosylglycerides and Galactosylceramides from the Microalga Isochrysis Galbana. J. Agric. Food Chem..

[50] Tocher D., Betancor M., Sprague M., Olsen R., Napier J. (2019). Omega-3 Long-Chain Polyunsaturated Fatty Acids, EPA and DHA: Bridging the Gap between Supply and Demand. Nutrients.

[51] Figueiredo A.R.P., da Costa E., Silva J., Domingues M.R., Domingues P. (2019). The Effects of Different Extraction Methods of Lipids from *Nannochloropsis oceanica* on the Contents of Omega-3 Fatty Acids. Algal Res..

[52] Siriwardhana N., Kalupahana N.S., Moustaid-Moussa N. (2012). Health Benefits of N-3 Polyunsaturated Fatty Acids. Eicosapentaenoic Acid and Docosahexaenoic Acid.

[53] Da Costa E., Silva J., Mendonça S.H., Abreu M.H., Domingues M.R. (2016). Lipidomic Approaches towards Deciphering Glycolipids from Microalgae as a Reservoir of Bioactive Lipids. Mar. Drugs.

[54] Liu T., Zhang L., Joo D., Sun S.C. (2017). NF-κB Signaling in Inflammation. Signal Transduct. Target. Ther..

[55] Natarajan K., Abraham P., Kota R., Isaac B. (2018). NF-κB-iNOS-COX2-TNF α Inflammatory Signaling Pathway Plays an Important Role in Methotrexate Induced Small Intestinal Injury in Rats. Food Chem. Toxicol..

[56] Robertson R.C., Guihéneuf F., Bahar B., Schmid M., Stengel D.B., Fitzgerald G.F., Ross R.P., Stanton C. (2015). The Anti-Inflammatory Effect of Algae-Derived Lipid Extracts on Lipopolysaccharide (LPS)-Stimulated Human THP-1 Macrophages. Mar. Drugs.

[57] Ulivi V., Lenti M., Gentili C., Marcolongo G., Cancedda R., Descalzi Cancedda F. (2011). Anti-Inflammatory Activity of Monogalactosyldiacylglycerol in Human Articular Cartilage in Vitro: Activation of an Anti-Inflammatory Cyclooxygenase-2 (COX-2) Pathway. Arthritis Res. Ther..

[58] Barros A., Pereira H., Campos J., Marques A., Varela J., Silva J. (2019). Heterotrophy as a Tool to Overcome the Long and Costly Autotrophic Scale-up Process for Large Scale Production of Microalgae. Sci. Rep..

[59] Ruiz J., Antequera T., Andres A.I., Petron M.J., Muriel E. (2004). Improvement of a Solid Phase Extraction Method for Analysis of Lipid Fractions in Muscle Foods. Anal. Chim. Acta.

[60] Alves E., Rey F., Melo T., Barros M.P., Domingues P., Domingues R. (2022). Bioprospecting Bioactive Polar Lipids from Olive (*Olea europaea* Cv. *Galega vulgar*) Fruit Seeds: LC-HR-MS/MS Fingerprinting and Sub-Geographic Comparison. Foods.

[61] Folch J., Lees M., Sloane G.H., Sloane Stanley G.H. (1957). A Simple Method For The Isolation and Purification of Total Lipids From Animal Tissues. J. Biol. Chem..

[62] Gortzi O., Lala S., Chinou I., Tsaknis J. (2007). Evaluation of the Antimicrobial and Antioxidant Activities of *Origanum dictamnus* Extracts before and after Encapsulation in Liposomes. Molecules.

[63] O’Brien J., Wilson I., Orton T., Pognan F. (2000). Investigation of the Alamar Blue (Resazurin) Fluorescent Dye for the Assessment of Mammalian Cell Cytotoxicity: Resazurin as a Cytotoxicity Assay. Eur. J. Biochem..

[64] Almeida A.S., Ferreira R.M.P., Silva A.M.S., Duarte A.C., Neves B.M., Duarte R.M.B.O. (2020). Structural Features and Pro-Inflammatory Effects of Water-Soluble Organic Matter in Inhalable Fine Urban Air Particles. Environ. Sci. Technol..

